# Clinical Significance of Tumor-Infiltrating Conventional and Plasmacytoid Dendritic Cells in Pancreatic Ductal Adenocarcinoma

**DOI:** 10.3390/cancers14051216

**Published:** 2022-02-26

**Authors:** Ioana Plesca, Iva Benešová, Carolin Beer, Ulrich Sommer, Luise Müller, Rebekka Wehner, Max Heiduk, Daniela Aust, Gustavo Baretton, Michael P Bachmann, Anja Feldmann, Jürgen Weitz, Lena Seifert, Adrian M Seifert, Marc Schmitz

**Affiliations:** 1 Institute of Immunology, Faculty of Medicine Carl Gustav Carus, TU Dresden, Fetscherstraße 74, 01307 Dresden, Germany; ioana-madalina.plesca@tu-dresden.de (I.P.); benesova.ivka@seznam.cz (I.B.); carolinbeer95@gmx.de (C.B.); luise.mueller1@tu-dresden.de (L.M.); rebekka.wehner@tu-dresden.de (R.W.); 2 Institute of Pathology, University Hospital Carl Gustav Carus, TU Dresden, Fetscherstraße 74, 01307 Dresden, Germany; ulrich.sommer2@uniklinikum-dresden.de (U.S.); daniela.aust@uniklinikum-dresden.de (D.A.); gustavo.baretton@uniklinikum-dresden.de (G.B.); 3 National Center for Tumor Diseases (NCT), University Hospital Carl Gustav Carus, TU Dresden, Fetscherstraße 74, 01307 Dresden, Germany; max.heiduk@uniklinikum-dresden.de (M.H.); m.bachmann@hzdr.de (M.P.B.); juergen.weitz@uniklinikum-dresden.de (J.W.); lena.seifert@uniklinikum-dresden.de (L.S.); adrian.seifert@uniklinikum-dresden.de (A.M.S.); 4 German Cancer Consortium (DKTK), Partner Site Dresden and German Cancer Research Center (DKFZ), Im Neuenheimer Feld 280, 69120 Heidelberg, Germany; 5 Department of Visceral, Thoracic and Vascular Surgery, University Hospital Carl Gustav Carus, TU Dresden, Fetscherstraße 74, 01307 Dresden, Germany; 6 Tumor Immunology, University Cancer Center (UCC), University Hospital Carl Gustav Carus, TU Dresden, Fetscherstraße 74, 01307 Dresden, Germany; 7 Department of Radioimmunology, Institute of Radiopharmaceutical Cancer Research, Helmholtz Center Dresden-Rossendorf, Bautzener Straße 400, 01328 Dresden, Germany; a.feldmann@hzdr.de

**Keywords:** pancreatic cancer, dendritic cells, tumor microenvironment, neoadjuvant chemotherapy

## Abstract

**Simple Summary:**

The tumor immune contexture plays a pivotal role for the clinical outcome of cancer patients and the efficacy of various treatment modalities. Dendritic cells (DCs) represent a major component of the tumor immune architecture that can either efficiently promote antitumor immunity or contribute to immunosuppression. Here, we investigated the frequency, spatial organization, and clinical significance of tumor-infiltrating conventional DCs type 1 (cDC1s) and type 2 (cDC2s) and plasmacytoid DCs (pDCs) in pancreatic ductal adenocarcinoma (PDAC). A higher frequency of whole tumor area (WTA)- and tumor stroma (TS)-infiltrating cDC1s, and of intraepithelial tumor-infiltrating cDC2s, was significantly associated with improved survival. Furthermore, a higher density of both WTA- and TS-infiltrating cDC1s and pDCs emerged as an independent prognostic factor for better survival. These results provide evidence that tumor-infiltrating DCs are associated with survival of PDAC patients and may support the design of novel DC-based immunotherapeutic strategies.

**Abstract:**

Dendritic cells (DCs) play a key role in the orchestration of antitumor immunity. Activated DCs efficiently enhance antitumor effects mediated by natural killer cells and T lymphocytes. Conversely, tolerogenic DCs essentially contribute to an immunosuppressive tumor microenvironment. Thus, DCs can profoundly influence tumor progression and clinical outcome of tumor patients. To gain novel insights into the role of human DCs in pancreatic ductal adenocarcinoma (PDAC), we explored the frequency, spatial organization, and clinical significance of conventional DCs type 1 (cDC1s) and type 2 (cDC2s) and plasmacytoid DCs (pDCs) in primary PDAC tissues. A higher density of whole tumor area (WTA)- and tumor stroma (TS)-infiltrating cDC1s was significantly associated with better disease-free survival (DFS). In addition, an increased frequency of intraepithelial tumor-infiltrating cDC2s was linked to better DFS and overall survival (OS). Furthermore, an increased density of WTA- and TS-infiltrating pDCs tended to improve DFS. Moreover, a higher frequency of WTA- and TS-infiltrating cDC1s and pDCs emerged as an independent prognostic factor for better DFS and OS. These findings indicate that tumor-infiltrating DCs can significantly influence the clinical outcome of PDAC patients and may contribute to the design of novel treatment options that target PDAC-infiltrating DCs.

## 1. Introduction

Pancreatic ductal adenocarcinoma (PDAC) is one of the most lethal cancers with a 5-year overall survival (OS) of 10% [1]. This very poor prognosis is caused by late disease detection, the absence of suitable biomarkers [2], and insufficient efficacy of current treatment options [3,4]. Despite the success of immunotherapy across solid tumors [5], the favorable outcome of this treatment modality remains limited in PDAC patients, with primary resection and chemotherapy still being the current standards of care [3,4,6]. Two of the main limitations of immunotherapy in PDAC patients are the mechanical barrier of the tumor surroundings, as well as the presence of immune cell populations with a predominantly immunosuppressive phenotype [3,7].

Desmoplasia, a characteristic hallmark of PDAC, is one of the major tumor escape mechanisms caused by activated pancreatic stellate cells (PSCs). These cells are the main source of fibrosis, which creates the mechanical barrier and, therefore, may hinder immune cell infiltration and may limit drug delivery. Furthermore, PSC’s proximity to cancer cells promotes tumor growth, invasion, and metastasis formation [8,9,10]. Another typical feature of PDAC is the immunosuppressive tumor microenvironment (TME) that is supported by PSCs through the secretion of cytokines, such as interleukin (IL)-10 and transforming growth factor (TGF)-β [9,11], and chemokines that attract regulatory T cells (Tregs) [12]. In addition, PSCs can produce galectin-1, which limits the cytotoxic properties of CD8^+^ T cells and promotes the differentiation of CD4^+^ T cells toward a T-helper (Th) 2 phenotype [9,11]. For these reasons, the TME of PDAC patients consists mainly of regulatory immune cells, such as Tregs, tumor-associated macrophages (TAMs) with dominant M2 phenotype, myeloid-derived suppressor cells (MDSCs), and immunosuppressive cytokines [7,13].

Dendritic cells (DCs) are professional antigen-presenting cells that play a critical role in the induction and maintenance of antitumor immunity. Due to their functional properties, DCs may profoundly influence tumor progression and the clinical outcome of cancer patients. Conventional DCs type 1 (cDC1s) and type 2 (cDC2s), and plasmacytoid DCs (pDCs) represent major subsets of human blood DCs that have been detected in various tumor entities. Human cDC1s characteristically express CD141 (BDCA-3), XCR1, and CLEC9A [14]. They display an extraordinary capacity to cross-present antigens and induce effective cytotoxic T lymphocyte (CTL) responses. In addition, activated cDC1s can produce significant amounts of IL-12 that favors the polarization of naïve CD4^+^ T lymphocytes into Th1 cells and promotes the cytokine release and cytotoxic capacity of natural killer (NK) cells [14,15,16,17]. When evaluating the potential clinical relevance of cDC1s, it has been reported that a higher expression of a cDC1-specific gene signature is correlated with favorable disease-free survival (DFS) of breast cancer patients [18]. Furthermore, higher expressions of cDC1-associated gene signatures were significantly associated with prolonged OS in patients with breast cancer, colorectal cancer, head and neck squamous cell carcinoma (HNSCC), lung adenocarcinoma, skin cutaneous melanoma, and ovarian cancer [19,20,21,22]. Moreover, a high frequency of melanoma-infiltrating CD40^+^ cDC1s predicted improved OS [23].

Identification of human cDC2s is based on high expression of various molecules, primarily CD1c and CLEC10A [14]. cDC2s display an extraordinary capacity to present MHC-II-associated antigens to CD4^+^ T lymphocytes and to promote the polarization of naïve CD4^+^ T cells into Th1, Th2, and Th17 cells. Activated cDC2s secrete large amounts of various cytokines, including tumor necrosis factor (TNF)-α, IL-1, IL-6, and IL-12 [24,25]. Higher expression of specific gene signatures for cDC2s was linked to better prognosis of patients with HNSCC, invasive breast carcinoma, lung adenocarcinoma, and skin cutaneous metastatic melanoma [20]. Conversely, a higher frequency of tumor-associated cDC2s was correlated with reduced OS in non-small cell lung cancer [26] and worse progression-free survival (PFS) of melanoma patients [23].

Human pDCs are mainly characterized by the expression of CD123, CD303 (BDCA-2), and CD304 (BDCA-4) [14]. pDCs present antigens with lower efficacy in comparison to cDCs, however, they are main producers of type I interferon upon stimulation. Interestingly, pDCs can directly kill cancer cells [27,28,29]. Enhanced expression of pDC-specific genes was associated with prolonged OS in HNSCC, papillary renal cell carcinoma, and lung adenocarcinoma [20]. Moreover, high levels of tumor-infiltrating CD86^+^ pDCs predicted prolonged PFS of melanoma patients [23]. More recently, we have shown that a higher density of tumor-associated pDCs is linked to improved PFS and OS of colon cancer patients [30]. In contrast, an enhanced frequency of tumor-infiltrating pDCs was associated with poor prognosis in breast cancer, hepatocellular cancer, melanoma, and ovarian cancer [31,32,33,34].

Previous studies have mainly explored the frequency of blood-circulating cDCs and pDCs and their association with clinicopathological characteristics of pancreatic cancer patients [35,36,37]. However, little is known about the potential clinical role of distinct PDAC-infiltrating human DC subsets. Here, we explored the density and spatial organization of PDAC-associated cDC1s, cDC2s, and pDCs by utilizing multiplex immunohistochemistry (mIHC). In addition, the frequency of tumor-infiltrating DC subsets was linked to the clinicopathological characteristics of PDAC patients to gain novel insights into their clinical relevance. Additionally, we evaluated the influence of neoadjuvant chemotherapy on the frequency of the PDAC-associated DC subsets.

## 2. Materials and Methods

### 2.1. Patient Samples

This is a retrospective study consisting of 58 PDAC patients treated with either neoadjuvant therapy or primary resection at the Department of Visceral, Thoracic, and Vascular Surgery of the University Hospital Carl Gustav Carus of Dresden between 2008 and 2015. This study received the approval of the institutional review board of the Faculty of Medicine of the TU Dresden (No EK446112017). Patients gave their written informed consent to participate in the study. Serial sections of formalin-fixed paraffin-embedded (FFPE) tumor tissues were stained with hematoxylin and eosin (H&E) for histologic assessment by an experienced pathologist. The clinical stage was determined by utilizing the pathological tumor-node-metastasis (pTNM) classification of the Union for International Cancer Control [38]. Table 1 summarizes the clinicopathological characteristics of PDAC patients.

### 2.2. Classical Immunohistochemistry

We conducted immunohistochemical stainings of the BDCA-2 molecule to investigate the presence, localization, and density of pDCs in the PDAC tissues of 58 patients as described previously [30]. Briefly, the FFPE tissue blocks, sectioned at a thickness of 3–5 μm, were first deparaffinized, rehydrated, and exposed to heat-induced antigen retrieval. Once preparation was completed, the samples were incubated with a goat anti-BDCA-2 antibody (1:200, polyclonal, R&D Systems, Minneapolis, MN, USA) overnight at 4 °C. A bridging step, consisting of a 10 min incubation with a mouse anti-goat antibody solution (Thermo Fisher Scientific, Rockford, IL, USA), preceded the labeling and visualization of the BDCA-2^+^ pDCs by using the alkaline phosphatase-based EnVision detection system (Dako, Glostrup, Denmark). At the end, all tissue slides were counterstained with Mayer’s hematoxylin (Merck, Darmstadt, Germany) and coverslipped with Aquatex mounting agent (Merck).

### 2.3. Multiplex Immunohistochemistry

To determine the presence, localization, and frequency of PDAC-associated cDC1s and cDC2s, we performed mIHC stainings in 40 tumor specimens. For this, we employed the Ventana Discovery Ultra Instrument (Roche, Basel, Switzerland) and the Opal multiplex reagents (Akoya Biosciences, Menlo Park, CA, USA) together with the Vectra 3 automated quantitative pathology imaging system (Akoya Biosciences). As for the classical immunohistochemical stainings, the samples were firstly prepared by deparaffinization and heat-mediated antigen retrieval using the cell conditioning 1 solution (Roche). Then, incubation of a primary antibody and a corresponding secondary antibody (OmniMap anti-mouse, anti-rabbit, or anti-mouse-HQ, ready-to-use, all from Roche) followed for 32 min and 12 min, respectively. In the case of the anti-mouse-HQ polymer, anti-HQ-HRP solution (Roche) was applied for another 12 min. After that, incubation with one of the tyramide signal amplification fluorophores (Opal 520, 540, 620, and 650, all from Akoya Biosciences) took place for 8 min. Heat treatment followed for 24 min to remove the primary and secondary antibodies. All these steps (excluding initial preparation) were repeated for each primary antibody in the panel, namely anti-CD1c (1:50, clone 2F4, Novus Biologicals, Littleton, Colorado, USA), anti-CLEC9A (1:100, clone EPR22324, Abcam, Cambridge, UK), anti-CLEC10A (1:75, polyclonal, Human Protein Atlas, Bromma, Sweden), and anti-PanCK (prediluted, clone AE1/AE3/PCK26, Roche). Finally, spectral DAPI (Akoya Biosciences) was added to the slides to counterstain the nuclei and a fluoromount medium (SouthernBiotech, Birmingham, Alabama, USA) was used for coverslipping.

### 2.4. Quantification of PDAC-Infiltrating cDC1s, cDC2s, and pDCs

All stained sections were whole scanned (×100 magnification) using the Vectra 3 automated quantitative pathology imaging system. The tumor-containing areas were marked in the Phenochart^TM^ software (Akoya Biosciences), following the delineation performed by an experienced pathologist in serial H&E slides. Then, a proportion of 25–50% of these tumor-containing regions was scanned (×200 magnification) for all patient slides for subsequent analysis. For the classical immunohistochemical stainings, positively stained pDCs were counted using the ImageJ software. These raw counts were used to determine the mean value, which was then converted into cell density. The analysis of mIHC stainings was performed using the inForm software (Akoya Biosciences). Following spectral unmixing, a semi-automatic approach was employed to teach the inForm software to segment the tissue areas (intraepithelial PanCK^+^ tumor region, stromal region, and non-tissue areas), delineate the cells, and phenotype them. For all analyses, we defined and used the whole tumor area (WTA) as the entire tumor region comprising both the intraepithelial tumor (IET) area and the tumor stroma (TS) area. Finally, the reliability of each algorithm was tested on a validation set of MSIs drawn from all patients, before applying them to the entire cohort.

### 2.5. Statistical Analysis

Mann–Whitney U test was performed to investigate the difference in DCs density at different tumor stages and for distinct therapy regimens, while the paired Wilcoxon test was used to compare DC frequencies between tumor localizations. Kaplan–Meier survival curves were utilized to visualize the differences in OS and DFS. OS was defined as the time period between surgery and death. DFS represents the time interval between surgery and disease recurrence. The patients were stratified into terciles (high, medium, and low infiltration), and only the high and low groups were compared. The significance was analyzed by log-rank test. A Cox proportional hazards regression model was implemented to explore the hazard ratio (HR) of DC infiltration in combination with several clinicopathological characteristics. Throughout the manuscript, the results are presented as mean value ± the standard error of the mean. All statistical analyses were performed using the R software, and values of *p* ≤ 0.05 were considered significant.

## 3. Results

### 3.1. cDC1s, cDC2s, and pDCs Infiltrate PDAC

**Figure 1:**
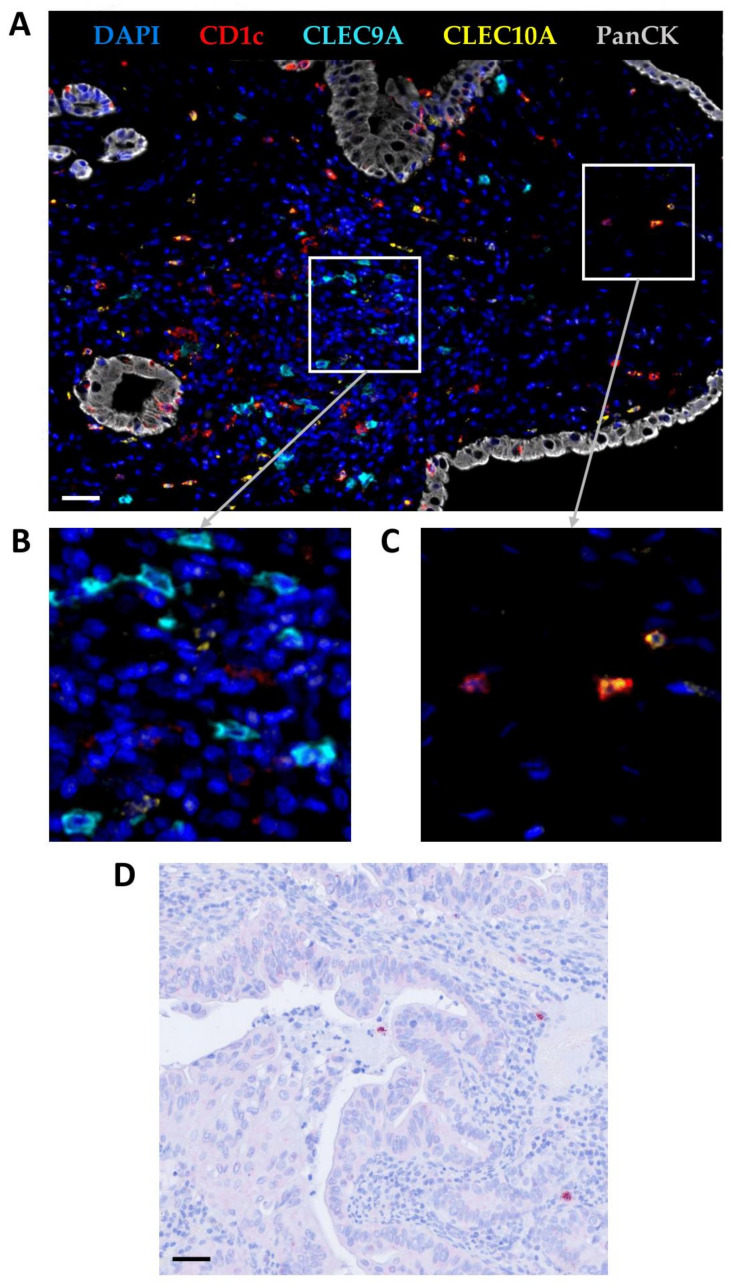
Conventional dendritic cells (DCs) type 1 (cDC1s) and type 2 (cDC2s), and plasmacytoid dendritic cells (pDCs) are cellular components of the pancreatic ductal adenocarcinoma (PDAC) immune architecture. (**A**–**D**) Immunohistochemical stainings were performed to assess the presence of tumor-infiltrating DCs. (**A**) Representative multiplex immunohistochemical staining of PDAC-infiltrating cDC1s (CLEC9A^+^ cells) and cDC2s (CD1c^+^CLEC10A^+^ cells) obtained by utilizing antibodies against CD1c (red), CLEC9A (yellow), CLEC10 (cyan), and PanCK (gray). Enlarged depictions of (**B**) cDC1s and (**C**) cDC2s. (**D**) Representative immunohistochemical staining of PDAC-infiltrating pDCs (BDCA-2^+^ cells). Original magnification of all images was ×200. Scale bars indicate 50 µm.

To evaluate the role of three major human blood DC subsets in PDAC, we investigated the presence and density of cDC1s, cDC2s, and pDCs in tissue specimens from PDAC patients with different clinicopathological characteristics (Table 1). The three DC subsets were detectable in all PDAC samples at varying frequencies (Figure 1A–D).

**Figure 2:**
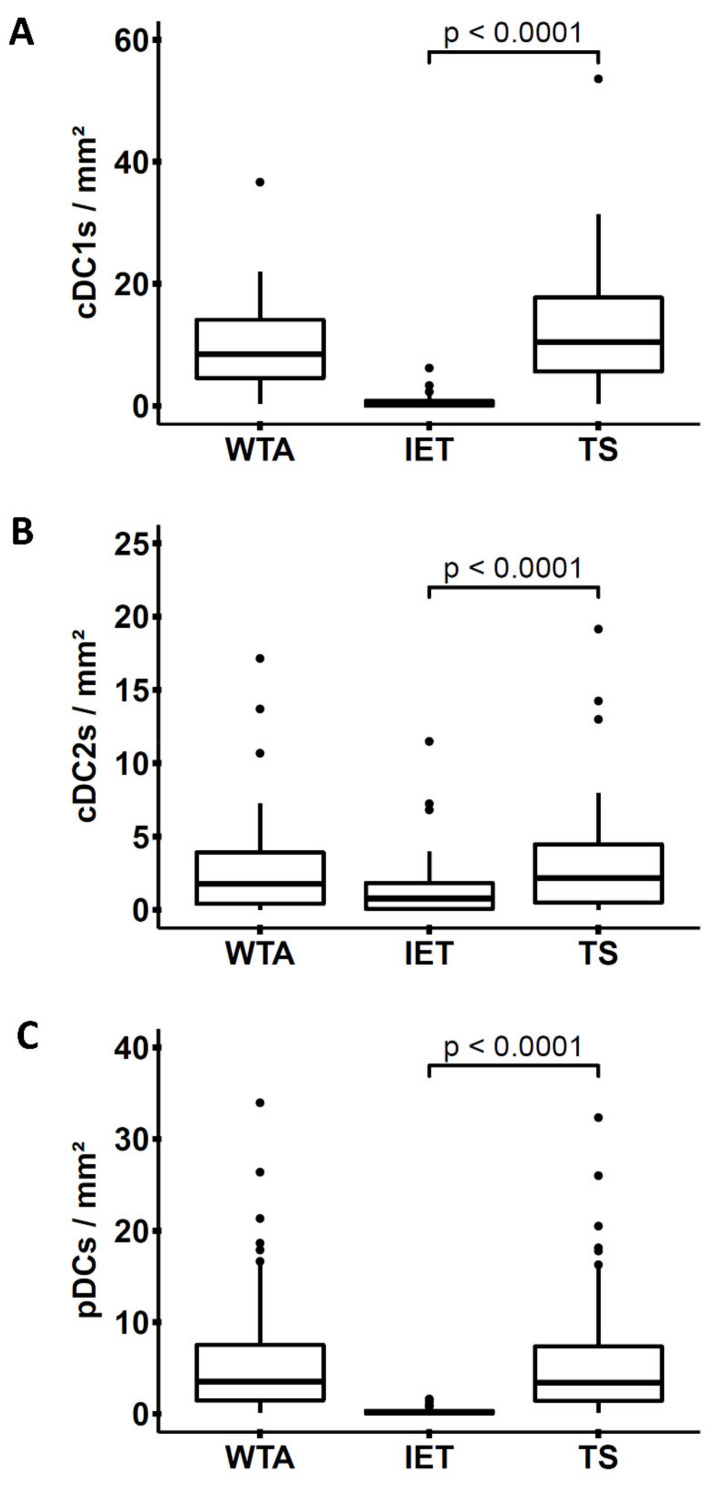
Frequency of pancreatic ductal adenocarcinoma (PDAC)-associated conventional dendritic cells (cDCs) and plasmacytoid dendritic cells (pDCs). Mean densities of PDAC-infiltrating (**A**) conventional DCs type 1 (cDC1s) (*n* = 40), (**B**) type 2 (cDC2s) (*n* = 40), and (**C**) plasmacytoid DCs (pDCs) (*n* = 58) were calculated for the whole tumor area (WTA), intraepithelial tumor area (IET), and tumor stroma (TS). Significance was determined by using paired Wilcoxon test, and *p* ≤ 0.05 was considered significant.

As depicted in Figure 2A–C, cDC1s were the most abundant DC subset in the WTA (9.84 ± 1.16 cDC1s/mm^2^) in comparison to cDC2s (2.98 ± 0.597 cDC2s/mm^2^) and pDCs (6.28 ± 0.917 pDCs/mm^2^). In addition, we observed a significantly higher density of cDC1s (12.6 ± 1.62 cDC1s/mm^2^), cDC2s (3.35 ± 0.662 cDC2s/mm^2^), and pDCs (6.04 ± 0.887 pDCs/mm^2^) in the TS compared to the IET area (0.679 ± 0.179 cDC1s/mm^2^, 1.51 ± 0.371 cDC2s/mm^2^, 0.246 ± 0.0436 pDCs/mm^2^). These findings provide evidence that cDC1s, cDC2s, and pDCs are cellular components of the PDAC immune contexture that are preferentially localized in the TS and may participate in the orchestration of antitumor immunity.

### 3.2. Levels of PDAC-Infiltrating cDCs Correlate with Favorable Pathological Tumor Features and with Increased Survival of Patients

**Figure 3:**
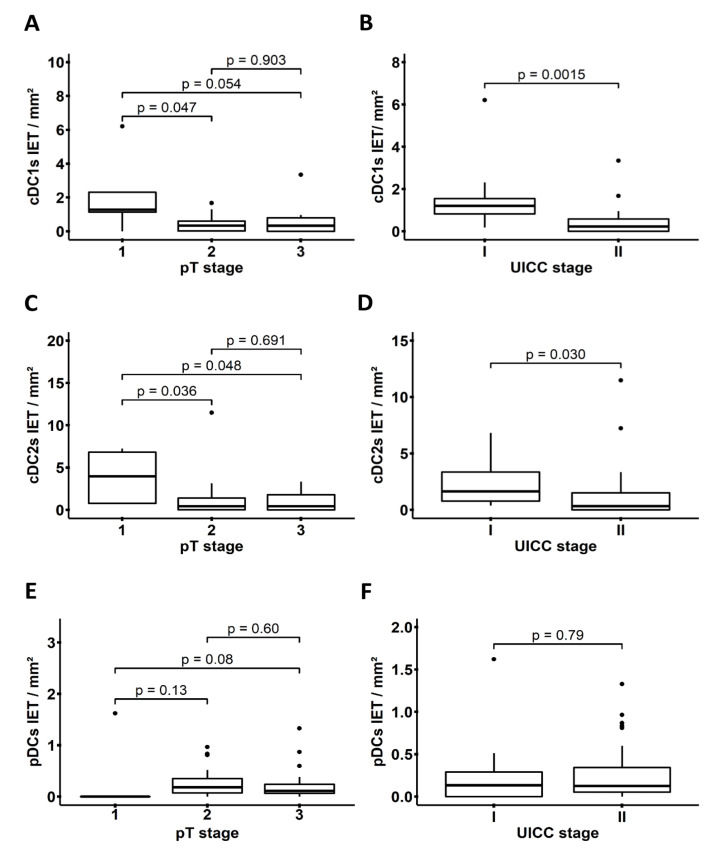
Frequency of intraepithelial tumor (IET) area-infiltrating dendritic cell (DC) subsets across distinct pathological tumor (pT) and Union for International Cancer Control (UICC) stages of pancreatic ductal adenocarcinoma (PDAC) patients. Boxplots show the density of IET-infiltrating conventional DCs type 1 (cDC1s) (*n* = 40), type 2 (cDC2s) (*n* = 40), and plasmacytoid DCs (pDCs) (*n* = 58) at different (**A**,**C**,**E**) tumor stages or (**B**,**D**,**F**) UICC stages. *p* values were calculated using the Mann–Whitney U test, and *p* ≤ 0.05 was considered significant.

Next, we evaluated whether the density of tumor-associated cDC1s, cDC2s, and pDCs correlates with relevant clinicopathological characteristics of PDAC patients. We observed a higher infiltration of cDC1s into the IET area of patients with early pT1 tumor stage vs. pT2 and pT3 stages (Figure 3A), and of patients classified as UICC stage I compared to UICC stage II (Figure 3B). Similarly, a significantly higher density of cDC2s was found within the IET area of patients with pT1 stage (Figure 3C) and UICC stage I (Figure 3D). In contrast to cDCs, a lower pDC infiltration was detected in pT1 stage tumors compared to pT3 stage tumors (Figure 3E), while the density of pDCs remained unchanged between UICC stage I and II (Figure 3F). Furthermore, no other significant differences were detected in the frequencies of DCs within the WTA (Figure S1A–F) or TS (Figure S2A–F) across different pT and UICC stages. However, there was a trend toward an increased infiltration of cDC2s into the WTA (Figure S1D) and TS (Figure S2D) of patients classified as UICC stage I vs. stage II.

**Figure 4:**
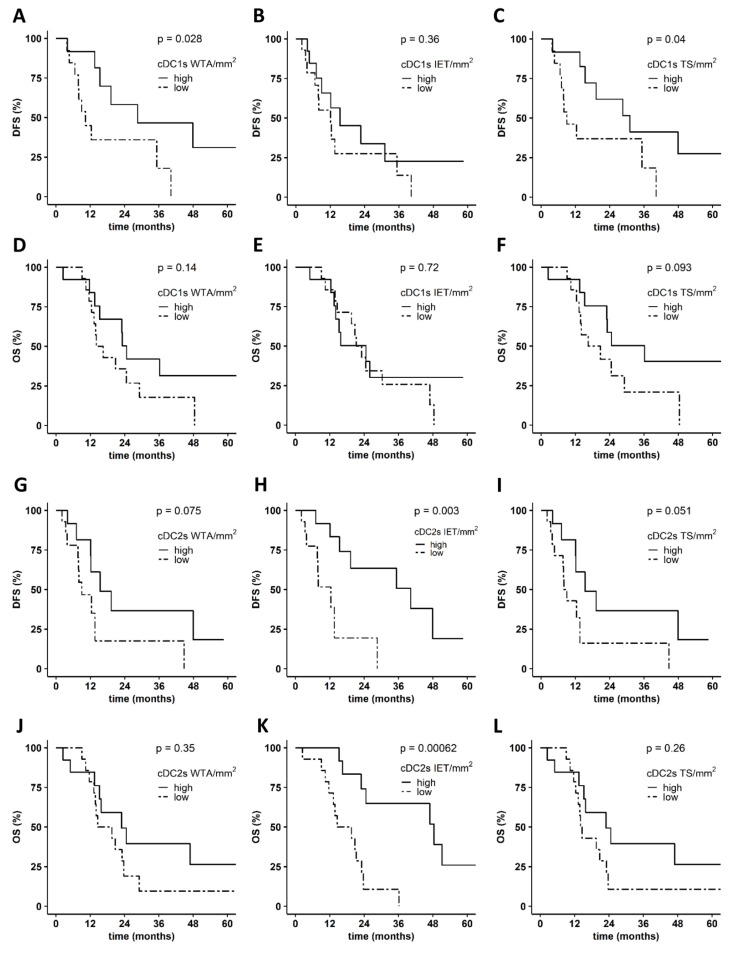
Association between the frequency of pancreatic ductal adenocarcinoma (PDAC)-infiltrating conventional dendritic cells (cDCs) and clinical outcome of patients. Kaplan–Meier curves illustrate the association between whole tumor area (WTA)-, intraepithelial tumor (IET)-, and tumor stroma (TS)-infiltrating cDCs type 1 (cDC1s) (*n* = 40) and (**A**–**C**) disease-free survival (DFS) or (**D**–**F**) overall survival (OS). Kaplan–Meier curves show the correlation between WTA-, IET-, and TS-infiltrating cDCs type 2 (cDC2s) (*n* = 40) and (**G**–**I**) DFS and (**J**–**L**) OS. Upper tercile (density > 2/3 of the patients in the analyzed cohort; solid line) and lower tercile (density ≤ 1/3 of patients in the analyzed cohort; dash line) were compared. *p* values were calculated using the log-rank test, and *p* ≤ 0.05 was considered significant.

**Figure 5:**
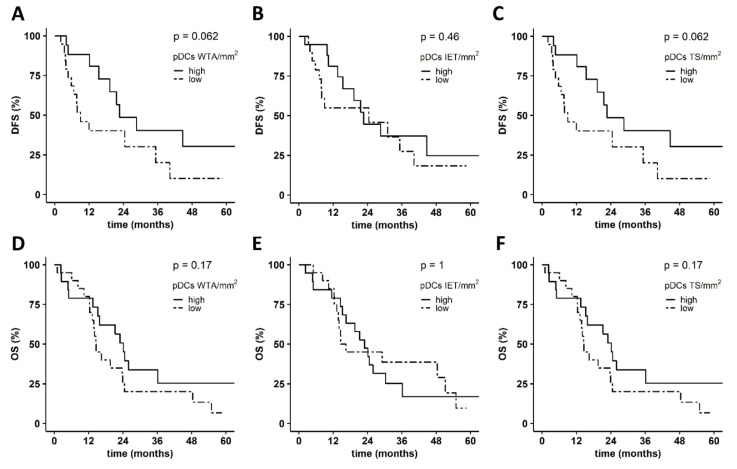
Association between the density of pancreatic ductal adenocarcinoma (PDAC)-infiltrating plasmacytoid dendritic cells (pDCs) and clinical outcome of patients. Kaplan–Meier curves illustrate the association between whole tumor area (WTA)-, intraepithelial tumor (IET)-, and tumor stroma (TS)-infiltrating pDCs (*n* = 58) and (**A**–**C**) disease-free survival (DFS) or (**D**–**F**) overall survival (OS). Upper tercile (density > 2/3 of the patients in the analyzed cohort; solid line) and lower tercile (density ≤ 1/3 of patients in the analyzed cohort; dash line) were compared. *p* values were calculated using the log-rank test, and *p* ≤ 0.05 was considered significant.

In further studies, we explored the correlation between the density of PDAC-infiltrating DC subsets and the clinical outcome of patients (Figure 4A–L). A higher density of WTA- and TS-infiltrating cDC1s was significantly associated with improved DFS in contrast to the IET-associated cDC1 frequency (Figure 4A–C). A trend for a correlation between an increased number of TS-infiltrating cDC1s and an improved OS was also observed, whereas the cDC1 density in the WTA and IET area did not influence OS (Figure 4D–F). As depicted in Figure 4G–L, a higher density of WTA-, IET-, and TS-infiltrating cDC2s was linked to better DFS and OS. Of note, the correlation between IET-infiltrating cDC2s and DFS or OS was significant (Figure 4H,K). When exploring the association between PDAC-infiltrating pDCs and the clinical outcome of patients, we found that an increased number of WTA- and TS-infiltrating pDCs tended to be correlated with an improved DFS, whereas high densities of IET-infiltrating pDCs did not impact DFS (Figure 5A–C). Additionally, pDCs infiltration did not significantly influence OS, irrespective of localization (Figure 5D–F). These results indicate that a higher frequency of WTA- and TS-infiltrating cDC1s, as well as of IET-infiltrating cDC2s, is significantly linked to better DFS.

Based on these findings, we applied a multivariate Cox proportional hazard model to evaluate the prognostic relevance of PDAC-infiltrating DCs by adjusting for several clinicopathological characteristics, including age, gender, and tumor stage. From this analysis, we observed that a higher frequency of WTA- and TS-infiltrating cDC1s represents an independent prognostic factor for both DFS and OS in PDAC patients (Table 2 and Table 3). Conversely, elevated levels of IET-infiltrating cDC1s and WTA-, IET-, and TS-infiltrating cDC2s did not show any prognostic significance for DFS and OS (Tables S1–S4). Furthermore, a higher density of WTA- and TS-infiltrating pDCs emerged as an independent prognostic factor for DFS, but not for OS (Table 4 and Table 5). A higher density of IET-infiltrating pDCs did not show any prognostic value for DFS and OS (Table S5). Altogether, these findings provide evidence that PDAC-infiltrating cDCs are associated with the clinical outcome of the patients, and cDC1s and pDCs may represent novel prognostic biomarkers.

### 3.3. Neoadjuvant Chemotherapy Does Not Influence the Frequency of DCs in PDAC

**Figure 6:**
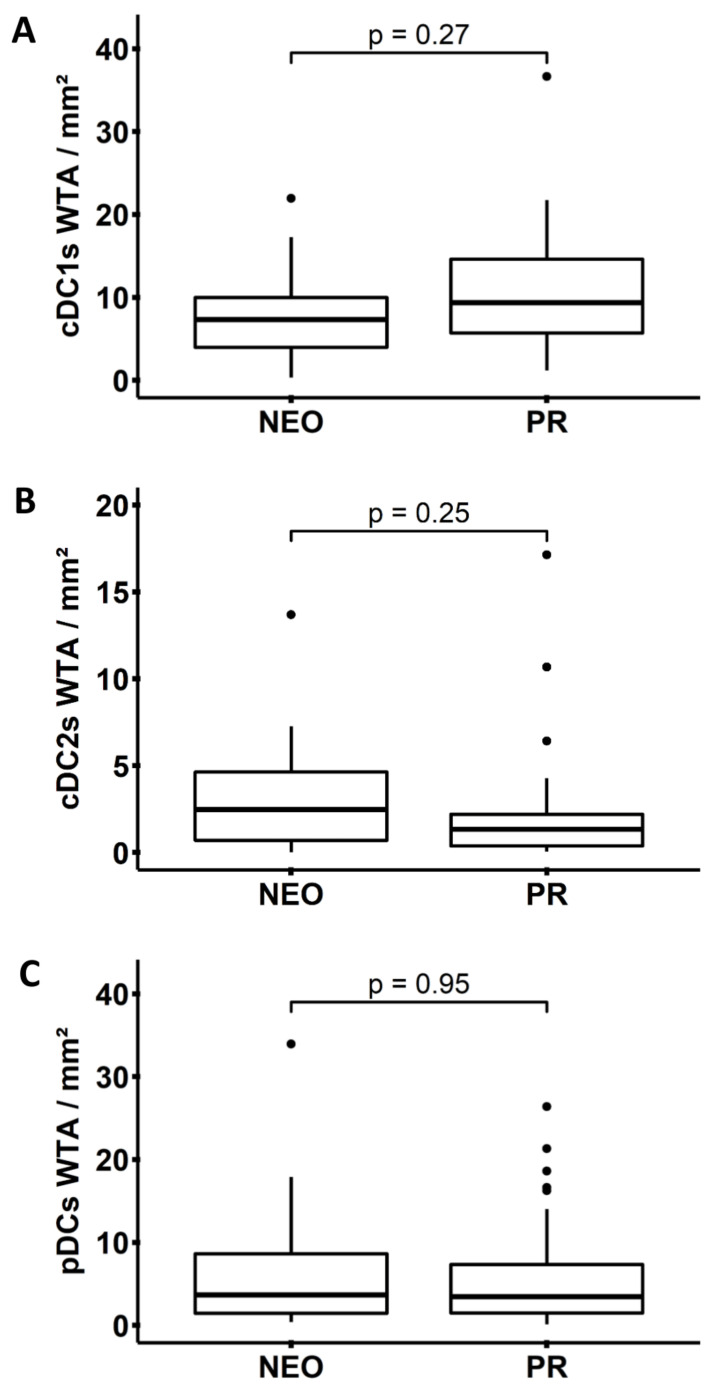
Effect of neoadjuvant chemotherapy (NEO) on the frequency of dendritic cells (DCs) within the whole tumor area (WTA) of pancreatic ductal adenocarcinoma (PDAC) patients. Boxplots show the frequencies of (**A**) conventional DCs type 1 (cDC1s) (*n* = 40), (**B**) type 2 (cDC2s) (*n* = 40), and (**C**) plasmacytoid DCs (pDCs) (*n* = 58) in patients treated with NEO or primary resection (PR). Significances were tested using the Mann–Whitney U test. *p* ≤ 0.05 was considered significant.

Recently, it has been demonstrated that chemotherapy can efficiently stimulate the antitumor immune response by triggering immunogenic cell death [39,40,41]. In contrast, chemotherapy can also induce immunosuppressive effects, including the increase in tumor-promoting MDSCs [41,42,43]. In addition, it has been reported that neoadjuvant chemotherapy results in an increased frequency of PDAC-infiltrating CD4^+^ and CD8^+^ T cells, while the density of Tregs and MDSCs decreases [44,45,46,47]. Following these findings, we explored the influence of neoadjuvant chemotherapy on the level of PDAC-associated DCs. As shown in Figure 6A–C, neoadjuvant chemotherapy did not significantly modulate the frequency of PDAC-infiltrating cDC1s, cDC2s, and pDCs.

## 4. Discussion

The tumor immune architecture plays a crucial role for the clinical outcome of cancer patients and influences the efficacy of various treatment modalities [48,49,50,51]. In PDAC, we and other groups have shown that elevated levels of tumor-infiltrating T lymphocytes favored the prognosis of PDAC patients [52,53,54,55,56]. The spatial distribution of these cells is critical, as it has been reported that close proximity of CD8^+^ T cells to tumor cells favored OS [52]. The clinical impact of PDAC-infiltrating T lymphocytes has also been demonstrated in a recent report, indicating that a high Immunoscore, which is characterized by elevated densities of CD3^+^ and CD8^+^ T cells in both the tumor center and invasive margin, is significantly associated with better disease-specific survival (DSS) and OS [57]. In contrast, we have observed that a high infiltration of LAG-3^+^ T cells is a negative independent prognostic factor for DFS [54]. In addition, an increased density of Tregs was linked to worse clinical outcome, while a high frequency of intratumoral PD-1^+^ Tregs was correlated with lymph node metastasis [53,58,59]. When investigating macrophages as another major component of the tumor immune contexture, it has been shown that they mainly polarize toward an M2 phenotype in PDAC [60]. Additionally, a meta-analysis of PDAC-associated macrophages revealed that M2 macrophages are linked to unfavorable survival [61]. So far, only a few studies have evaluated other PDAC-infiltrating immune cell populations. Among them, it has been reported that high neutrophil counts negatively impact OS [55], whereas increased B-cell densities are associated with better survival [62]. The presence of B cells in the tumor-associated tertiary lymphoid structures, but not in the TS, favored patients’ clinical outcome [63].

In contrast to T cells and macrophages, studies investigating the frequency, spatial organization, and clinical relevance of distinct human DC subsets in PDAC are very limited. DCs can essentially contribute to innate and adaptive antitumor immunity. In contrast, they can act as tolerogenic DCs by inhibiting tumor-directed immune responses. Hence, DCs may profoundly influence tumor development and progression, as well as clinical outcome. To gain novel insights into the role of DCs in pancreatic cancer, recent studies have used murine models. Thus, it has been shown that tumor-infiltrating cDCs are rare and dysfunctional and that enhancing the influx and activation of cDCs in established PDAC restores antitumor T-cell immunity, resulting in disease stabilization [64]. Furthermore, it has been reported that murine cDC1s are systemically dysregulated early in pancreatic cancer and that apoptosis of cDC1s mediated by IL-6 essentially contributes to this effect [65]. In addition, it has been demonstrated that Tregs promote a tolerogenic phenotype of PDAC-associated CD11c^+^ DCs, leading to an insufficient activation of CD8^+^ CTLs [66]. Meyer et al. observed that granulocyte colony-stimulating factor produced by pancreatic cancer cells inhibits interferon regulatory factor-8 expression in cDCs progenitors, leading to reduced cDC1 generation and impaired antitumor CD8^+^ T cell responses [67]. Another recent study has reported that CD11b^+^CD11c^+^MHC-II^+^CD24^+^CD64^low^F4/80^low^ DCs are an important component of PDAC-associated metastases that induce the expansion of Tregs and promote metastasis formation [68].

To explore the role of human DCs in PDAC, previous studies mainly evaluated the frequency and potential clinical impact of blood-circulating cDCs and pDCs. PDAC patients have reduced proportions of cDCs and pDCs in the blood compared to healthy donors [35,36,37,65]. In addition, it has been demonstrated that PDAC patients who survived longer had a higher percentage of circulating cDCs compared to those patients with shorter survival [35,36,37]. So far, only a few studies have investigated the density and clinical relevance of PDAC-infiltrating human DCs. When utilizing public databases, it has been shown that the density of activated DCs in PDAC tissues is significantly higher compared to para-tumor tissues [69]. Additionally, PDAC patients with early-stage tumors had significantly higher levels of tumor-infiltrating fascin^+^ DCs compared to more advanced tumor stages. Moreover, an elevated density of tumor-infiltrating fascin^+^ DCs was linked to improved survival of PDAC patients [37]. To gain novel insights into the clinical relevance of distinct human DC subsets in PDAC, we investigated the density and spatial organization of PDAC-associated cDC1s, cDC2s, and pDCs by utilizing mIHC and by evaluating their association with clinicopathological parameters. All three DC subsets were detectable in PDAC tissues and were preferentially located in the TS. The preferential localization of DCs in the TS was also previously shown in ovarian carcinoma, where mature DC-LAMP^+^ cells were mainly found in TS rather than in the IET area [70], as well as in breast cancer, where cDC1s were primarily located in the TS, close to CD8^+^ T lymphocytes [20]. Similarly, CD11c^+^ cDCs cells were significantly increased in the TS compared to the IET area in melanoma metastases [71]. In further experiments, we evaluated the clinical impact of the spatial organization of the three DC subsets within the PDAC tissues, given that the spatial distribution emerged as an important factor influencing patients’ prognosis in previous studies. High densities of TS-infiltrating CD4^+^ T cells favored DSS of non-small-cell lung carcinoma patients, whereas their frequency in the tumor islands did not have any effect on DSS [72]. In contrast, an increased frequency of tumor island-associated CD3^+^ T cells was linked to improved relapse-free survival in triple negative breast cancer [73]. Here, we showed that in contrast to IET-infiltrating cDC1s, a higher density of TS-associated cDC1s was significantly correlated with prolonged DFS. These findings provide evidence that the density and spatial organization of PDAC-associated cDC1s, cDC2s, and pDCs play an important role for the clinical outcome of PDAC patients.

Taken together, we found that cDC1s, cDC2s, and pDCs were present in primary PDAC tissues at varying frequencies and were mainly located in the TS. Higher levels of IET-infiltrating cDC1s and cDC2s were detectable in pT1 and UICC I stages compared to higher stages. Moreover, elevated infiltration of cDC1s within the WTA and TS was significantly correlated with improved DFS. We also observed that an increased frequency of IET-infiltrating cDC2s was linked to better DFS and OS when performing Kaplan–Meier analysis, while a higher level of WTA- and TS-infiltrating pDCs tended to improve DFS. Moreover, a higher frequency of both WTA- and TS-infiltrating cDC1s and pDCs emerged as an independent prognostic factor for better DFS and OS. Additionally, neoadjuvant chemotherapy did not significantly influence the density of PDAC-infiltrating DCs.

## 5. Conclusions

These novel findings indicate that cDC1s, cDC2s, and pDCs, as major human blood DC subsets, are components of the PDAC immune landscape. In addition, our results provide evidence that tumor-infiltrating DCs are associated with the survival of PDAC patients and that their density and spatial organization play an important role for the clinical outcome. PDAC-infiltrating cDC1s and pDCs may represent new independent prognostic markers for improved survival. Our data may also have implications for the generation of new treatment strategies that target PDAC-infiltrating DCs.

## Figures and Tables

**Table 1:** Clinicopathologic characteristics of pancreatic ductal adenocarcinoma (PDAC) patients.

	*n* = 58 *n* (%)
**Age**	
Median (Range)	67.14 (47–79)
**Gender**	
Male	30 (52)
Female	28 (48)
**pT Stage**	
1	5 (9)
2	33 (57)
3	20 (34)
**pN Stage**	
0	35 (60)
1	23 (40)
**pM Stage**	
0	58 (100)
**UICC Stage**	
I	8 (14)
II	50 (86)
**Neoadjuvant Chemotherapy**	
Yes	21 (36)
No	37 (64)

Abbreviations: pT: pathological tumor, pN: pathological node, pM: pathological metastasis, UICC: Union for International Cancer Control.

**Table 2:** Higher densities of whole tumor area (WTA)-infiltrating conventional dendritic cells type 1 (cDC1s) are associated with favorable disease-free survival (DFS) and overall survival (OS). Hazard ratios (HR) and 95% confidence intervals (CI) are shown.

DFS–WTA	*n*	HR	95% CI	*p* -Value
**cDC1s**	40	0.89	0.82–0.97	0.006 **
**Age**	40	0.92	0.86–0.99	0.017 *
**Female vs. male**	16 vs. 24	1.69	0.64–4.47	0.294
**pT stage**				
pT2 vs. pT1	22 vs. 5	2.04	0.52–8.02	0.306
pT3 vs. pT1	13 vs. 5	2.26	0.52–9.77	0.273
**pN stage**				
pN1 vs. pN0	14 vs. 26	1.79	0.68–4.70	0.239
**OS–WTA**				
**cDC1s**	40	0.92	0.85–0.98	0.016 *
**Age**	40	0.96	0.91–1.01	0.094
**Female vs. male**	16 vs. 24	1.70	0.74–3.93	0.214
**pT stage**				
pT2 vs. pT1	22 vs. 5	5.41	0.67–43.51	0.113
pT3 vs. pT1	13 vs. 5	3.77	0.46–31.09	0.217
**pN stage**				
pN1 vs. pN0	14 vs. 26	2.88	1.28–6.50	0.011 *

Abbreviations: pT: pathological tumor, pN: pathological node. *p* ≤ 0.05 was considered significant. * *p* ≤ 0.05, ** *p* ≤ 0.01.

**Table 3:** Higher frequencies of tumor stroma (TS)-infiltrating conventional dendritic cells type 1 (cDC1s) are correlated with better disease-free survival (DFS) and overall survival (OS). Hazard ratio (HR) and 95% confidence intervals (CI) are shown.

DFS–TS	*n*	HR	95% CI	*p* -Value
**cDC1s**	40	0.91	0.86–0.97	0.005 **
**Age**	40	0.91	0.85–0.98	0.013 *
**Female vs. male**	16 vs. 24	1.84	0.67–5.04	0.237
**pT stage**				
pT2 vs. pT1	22 vs. 5	1.88	0.49–7.26	0.361
pT3 vs. pT1	13 vs. 5	1.98	0.46–8.55	0.36
**pN stage**				
pN1 vs. pN0	14 vs. 26	1.83	0.70–4.84	0.22
**OS–TS**				
**cDC1s**	40	0.93	0.88–0.98	0.012 *
**Age**	40	0.95	0.90–1.01	0.086
**Female vs. male**	16 vs. 24	1.70	0.73–3.96	0.22
**pT stage**				
pT2 vs. pT1	22 vs. 5	4.91	0.62–39.22	0.133
pT3 vs. pT1	13 vs. 5	3.24	0.39–26.86	0.277
**pN stage**				
pN1 vs. pN0	14 vs. 26	2.94	1.30–6.69	0.01 **

Abbreviations: pT: pathological tumor, pN: pathological node. *p* ≤ 0.05 was considered significant. * *p* ≤ 0.05, ** *p* ≤ 0.01.

**Table 4:** Higher densities of whole tumor area (WTA)-infiltrating plasmacytoid dendritic cells (pDCs) influence disease-free survival (DFS) but not overall survival (OS). Hazard ratio (HR) and 95% confidence intervals (CI) are shown. *p* ≤ 0.05 was considered significant.

DFS–WTA	*n*	HR	95% CI	*p* -Value
**pDCs**	58	0.90	0.82–0.98	0.018 *
**Age**	58	0.94	0.90–0.99	0.01 *
**Female vs. male**	28 vs. 30	1.38	0.61–3.12	0.445
**pT stage**				
pT2 vs. pT1	33 vs. 5	1.74	0.47–6.46	0.405
pT3 vs. pT1	20 vs. 5	2.97	0.74–11.91	0.124
**pN stage**				
pN1 vs. pN0	35 vs. 23	3.08	1.35–7.03	0.008 **
**OS–WTA**				
**pDCs**	58	0.95	0.89–1.0	0.135
**Age**	58	0.98	0.94–1.0	0.28
**Female vs. male**	28 vs. 30	1.21	0.60–2.4	0.587
**pT stage**				
pT2 vs. pT1	33 vs. 5	4.81	0.63–36.9	0.13
pT3 vs. pT1	20 vs. 5	4.87	0.61–38.8	0.135
**pN stage**				
pN1 vs. pN0	35 vs. 23	3.23	1.65–6.3	<0.001 ***

Abbreviations: pT: pathological tumor, pN: pathological node. * *p* ≤ 0.05, ** *p* ≤ 0.01, *** *p* ≤ 0.001.

**Table 5:** Higher densities of tumor stroma (TS)-infiltrating plasmacytoid dendritic cells (pDCs) influence disease-free survival (DFS) but not overall survival (OS). Hazard ratio (HR) and 95% confidence interval (CI) are shown. *p* ≤ 0.05 was considered significant.

DFS–TS	*n*	HR	95% CI	*p* Value
**pDCs**	58	0.89	0.81–0.98	0.017 *
**Age**	58	0.94	0.90–0.99	0.01 *
**Female vs. male**	28 vs. 30	1.38	0.61–3.13	0.445
**pT stage**				
pT2 vs. pT1	33 vs. 5	1.74	0.47–6.46	0.405
pT3 vs. pT1	20 vs. 5	2.98	0.74–11.95	0.124
**pN stage**				
pN1 vs. pN0	35 vs. 23	3.08	1.35–7.04	0.008 **
**OS–TS**				
**pDCs**	58	0.95	0.89–1.0	0.144
**Age**	58	0.98	0.94–1.0	0.276
**Female vs. male**	28 vs. 30	1.21	0.60–2.4	0.588
**pT stage**				
pT2 vs. pT1	33 vs. 5	4.79	0.63–36.7	0.131
pT3 vs. pT1	20 vs. 5	4.82	0.61–38.3	0.137
**pN stage**				
pN1 vs. pN0	35 vs. 23	3.21	1.65–6.3	<0.001 ***

Abbreviations: pT: pathological tumor, pN: pathological node. * *p* ≤ 0.05, ** *p* ≤ 0.01, *** *p* ≤ 0.001.

## Data Availability

The data presented in this study are available on request from the corresponding author.

## Supplementary Materials

The following supporting information can be downloaded at: https://www.mdpi.com/article/10.3390/cancers14051216/s1, Figure S1: Frequency of whole tumor area (WTA)-infiltrating dendritic cell (DC) subsets across distinct pathological tumor (pT) and Union for International Cancer Control (UICC) stages of pancreatic ductal adenocarcinoma (PDAC) patients, Figure S2: Frequency of tumor stroma (TS)-infiltrating dendritic cell (DC) subsets across distinct pathological tumor (pT) and Union for International Cancer Control (UICC) stages of pancreatic ductal adenocarcinoma (PDAC) patients, Table S1: Higher densities of intraepithelial tumor (IET)-infiltrating conventional dendritic cells type 1 (cDC1s) do not influence disease-free survival (DFS) and overall survival (OS), Table S2: Higher frequencies of whole tumor area (WTA)-infiltrating conventional dendritic cells type 2 (cDC2s) do not alter disease-free survival (DFS) and overall survival (OS), Table S3: Higher densities of intraepithelial tumor (IET)-infiltrating conventional dendritic cells type 2 (cDC2s) do not modulate disease-free survival (DFS) and overall survival (OS), Table S4: Higher frequencies of tumor stroma (TS)-infiltrating conventional dendritic cells type 2 (cDC2s) do not influence disease-free survival (DFS) and overall survival (OS), Table S5: Higher densities of intraepithelial tumor (IET)-infiltrating plasmacytoid dendritic cells (pDCs) do not alter disease-free survival (DFS) and overall survival (OS).
