# Supplementary material for: Clinical Significance of Tumor-Infiltrating Conventional and Plasmacytoid Dendritic Cells in Pancreatic Ductal Adenocarcinoma
Source: Cancers (Basel). 2022 Feb 26;14(5):1216. doi: 10.3390/cancers14051216 (PMC8909898; doi:10.3390/cancers14051216)
Supplement: Supplementary file 1 [file cancers-14-01216-s001.zip › Supplementary Material - Legends.pdf]

Figure S1 Frequency of whole tumor area (WTA)-infiltrating dendritic cell (DC) subsets across distinct pathological tumor (pT) and Union for International Cancer Control (UICC) stages of pancreatic ductal adenocarcinoma (PDAC) patients. Boxplots depict the density of WTA-infiltrating conventional DCs type 1 (cDC1s) (n = 40), type 2 (cDC2s) (n = 40), and plasmacytoid DCs (pDCs) (n = 58) across different (**A, C, E**) pT stages and (**B, D, F**) UICC stages. p values were calculated using the Mann-Whitney U test and $p \leq 0.05$ was considered significant.

Figure S2 Frequency of tumor stroma (TS)-infiltrating dendritic cell (DC) subsets across distinct pathological tumor (pT) and Union for International Cancer Control (UICC) stages of pancreatic ductal adenocarcinoma (PDAC) patients. Boxplots show the frequency of TS- infiltrating conventional DCs type 1 (cDC1s) (n = 40), type 2 (cDC2s) (n = 40), and plasmacytoid DCs (pDCs) (n = 58) across different (**A, C, E**) pT stages or (**B, D, F**) UICC stages. p values were calculated using the Mann-Whitney U test and $p \leq 0.05$ was considered significant.

Table S1 Higher densities of intraepithelial tumor (IET)-infiltrating conventional dendritic cells type 1 (cDC1s) do not influence disease-free survival (DFS) and overall survival (OS). Hazard ratios (HR) and 95% confidence intervals (CI) are shown. Abbreviations: pT: pathological tumor, pN: pathological node. $p \leq 0.05$ was considered significant. * $p < 0.05$

Table S2 Higher frequencies of whole tumor area (WTA)-infiltrating conventional dendritic cells type 2 (cDC2s) do not alter disease-free survival (DFS) and overall survival (OS). Hazard ratios (HR) and 95% confidence intervals (CI) are shown. Abbreviations: pT: pathological tumor, pN: pathological node. $p \leq 0.05$ was considered significant. * $p < 0.05$

Table S3 Higher densities of intraepithelial tumor (IET)-infiltrating conventional dendritic cells type 2 (cDC2s) do not modulate disease-free survival (DFS) and overall survival (OS). Hazard ratios (HR) and 95% confidence intervals (CI) are shown. Abbreviations: pT: pathological tumor, pN: pathological node. $p \leq 0.05$ was considered significant. * $p < 0.05$

Table S4 Higher frequencies of tumor stroma (TS)-infiltrating conventional dendritic cells type 2 (cDC2s) do not influence disease-free survival (DFS) and overall survival (OS). Hazard ratios (HR) and 95% confidence intervals (CI) are shown. Abbreviations: pT: pathological tumor, pN: pathological node. $p \leq 0.05$ was considered significant. * $p < 0.05$

Table S5 Higher densities of intraepithelial tumor (IET)-infiltrating plasmacytoid dendritic cells (pDCs) do not alter disease-free survival (DFS) and overall survival (OS). Hazard ratios (HR) and 95% confidence intervals (CI) are shown. Abbreviations: pT: pathological tumor, pN: pathological node. $p \leq 0.05$ was considered significant. * $p < 0.05$, ** $p < 0.01$, *** $p < 0.001$
