# Supplementary figures and images for: Clinical Significance of Tumor-Infiltrating Conventional and Plasmacytoid Dendritic Cells in Pancreatic Ductal Adenocarcinoma
Source: Cancers (Basel). 2022 Feb 26;14(5):1216. doi: 10.3390/cancers14051216 (PMC8909898; doi:10.3390/cancers14051216)

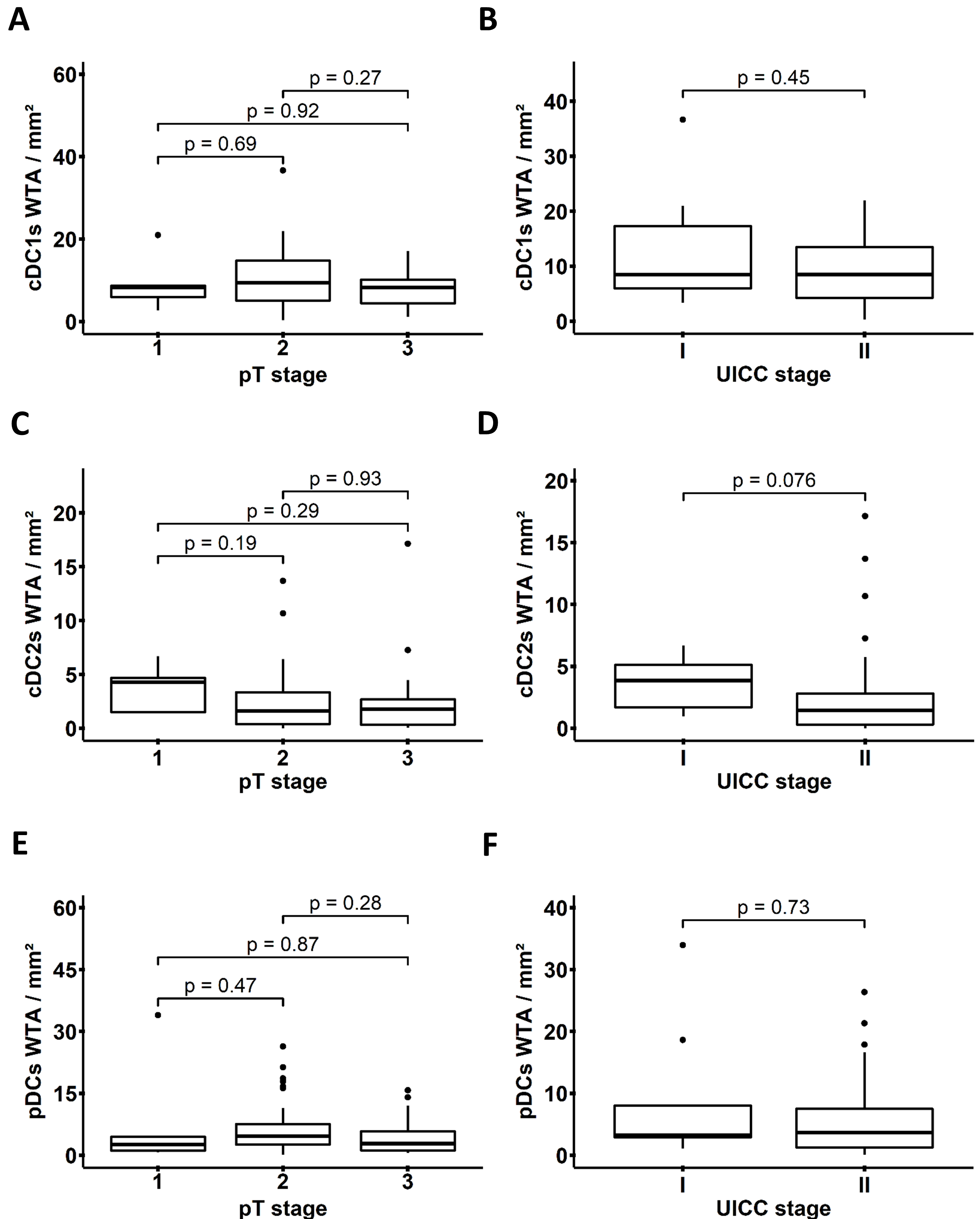

Supplement: Supplementary file 1 [file cancers-14-01216-s001.zip › Figure S1.tif]

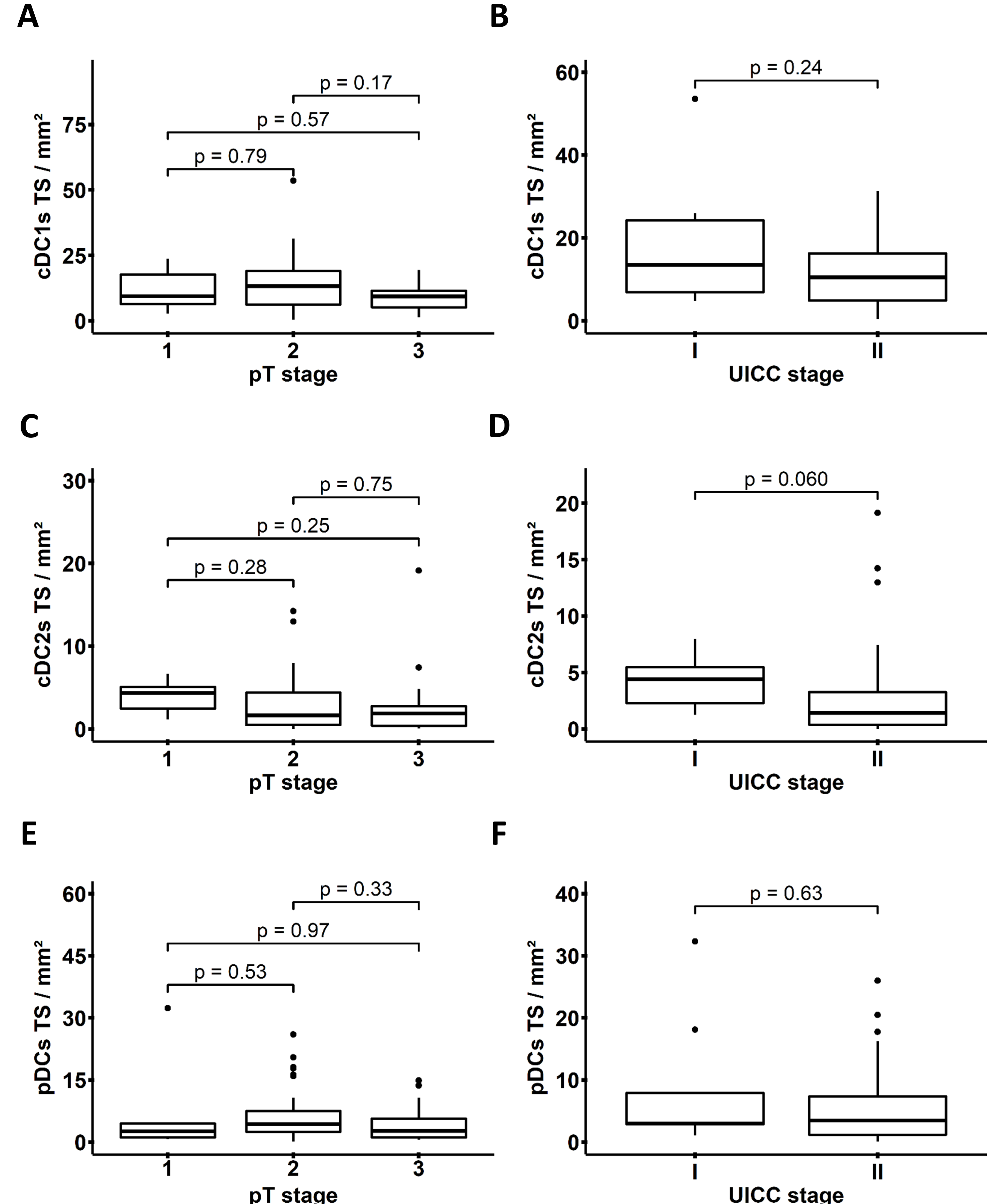

Supplement: Supplementary file 1 [file cancers-14-01216-s001.zip › Figure S2.tif]

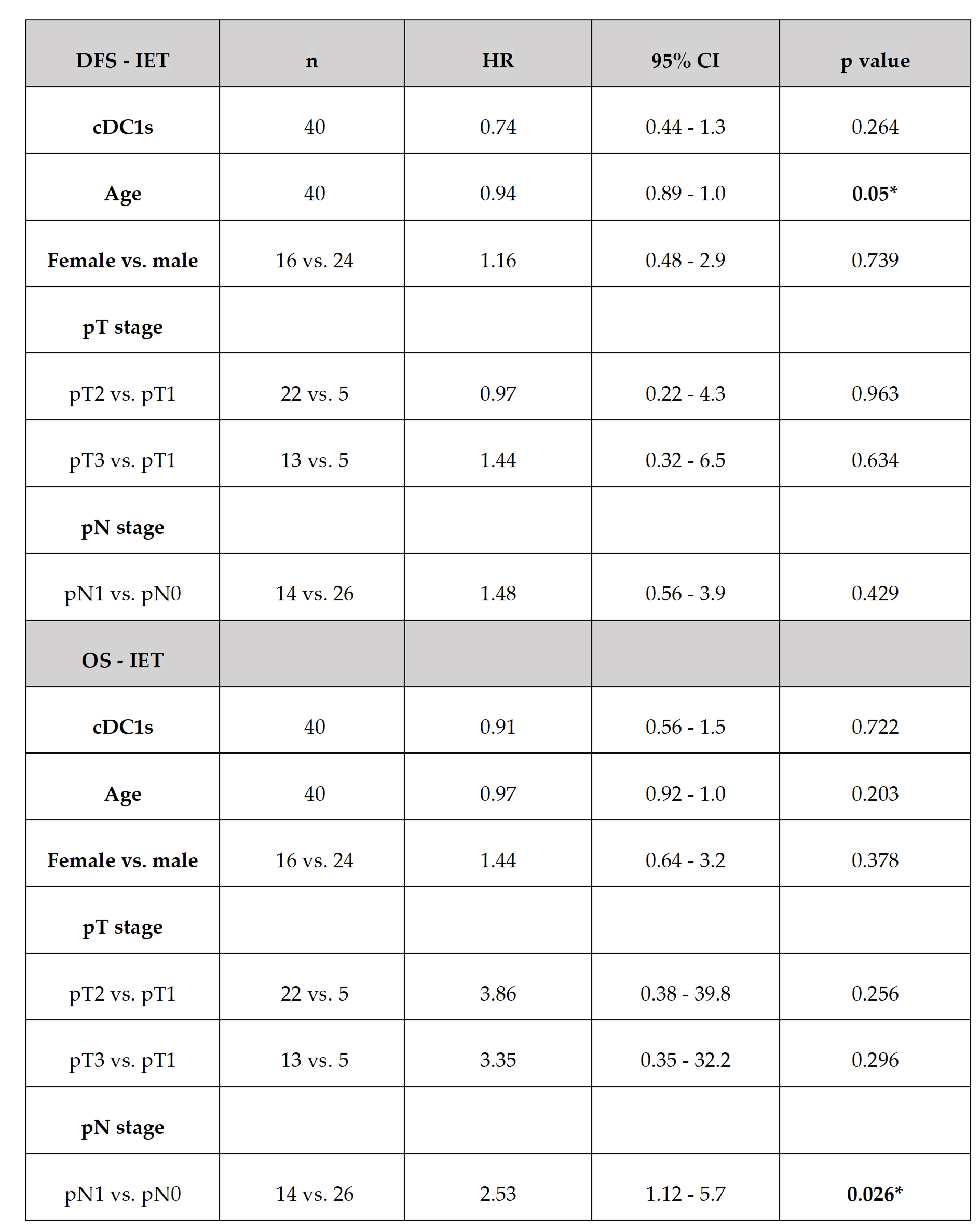

Supplement: Supplementary file 1 [file cancers-14-01216-s001.zip › Table S1.tif]

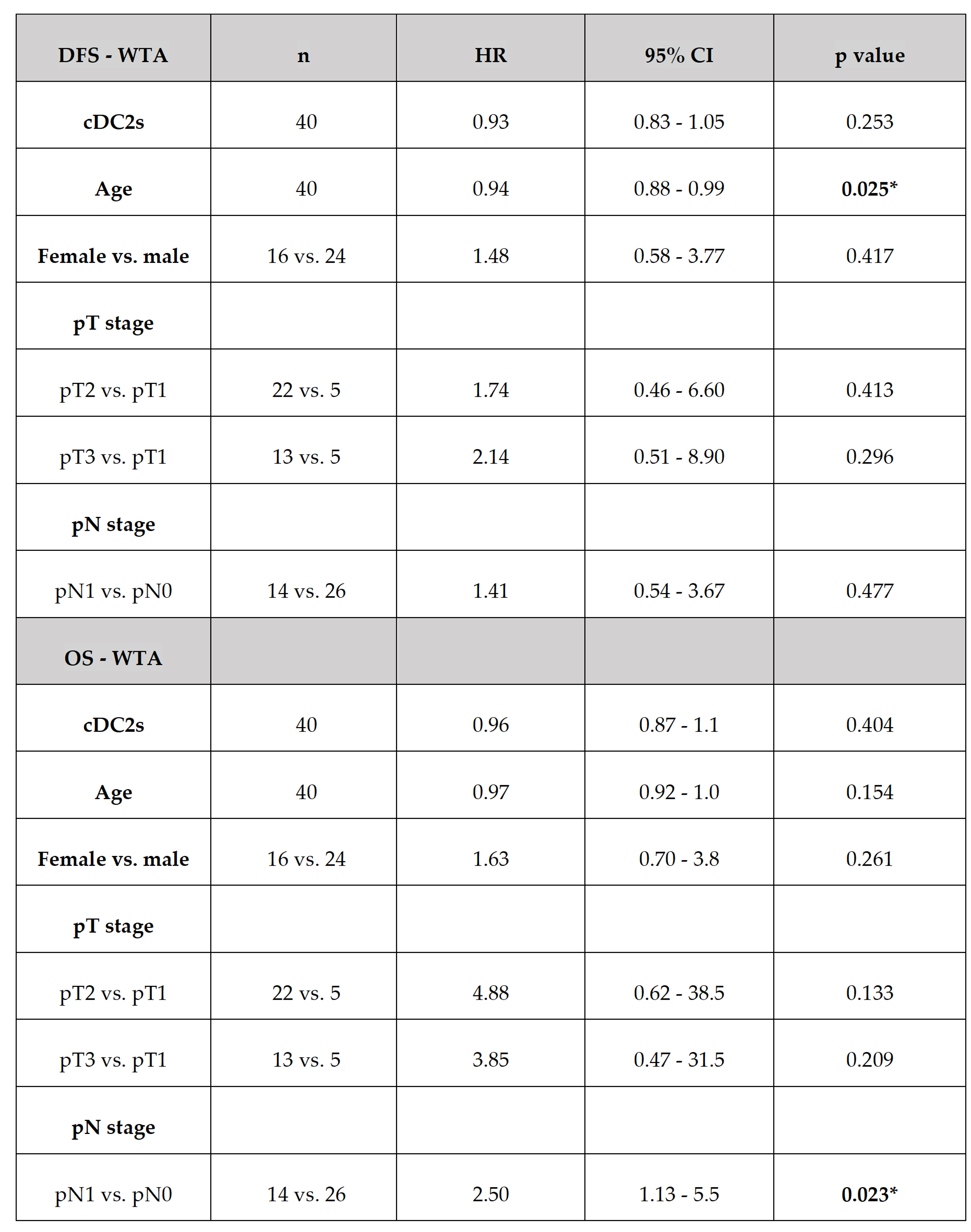

Supplement: Supplementary file 1 [file cancers-14-01216-s001.zip › Table S2.tif]

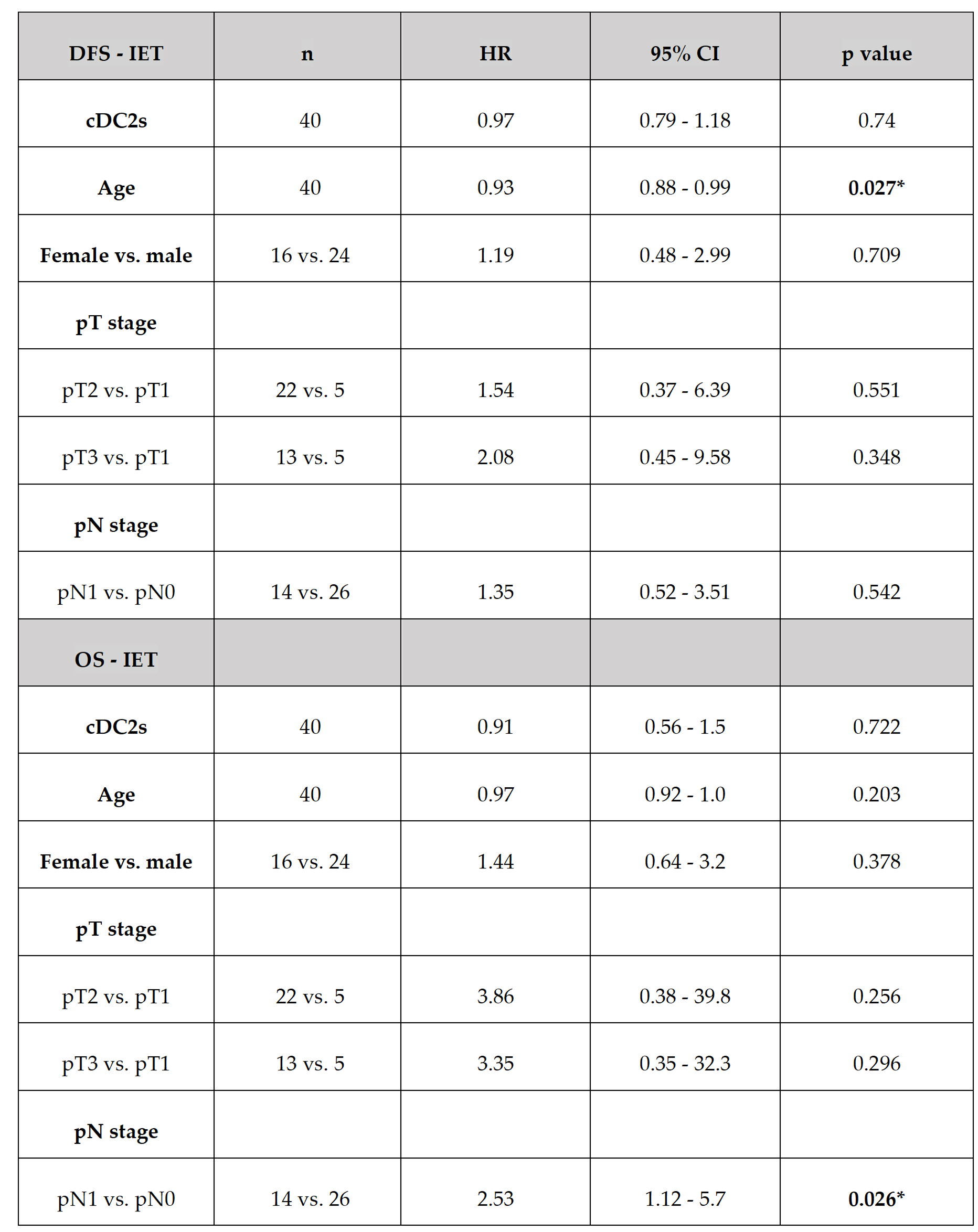

Supplement: Supplementary file 1 [file cancers-14-01216-s001.zip › Table S3.tif]

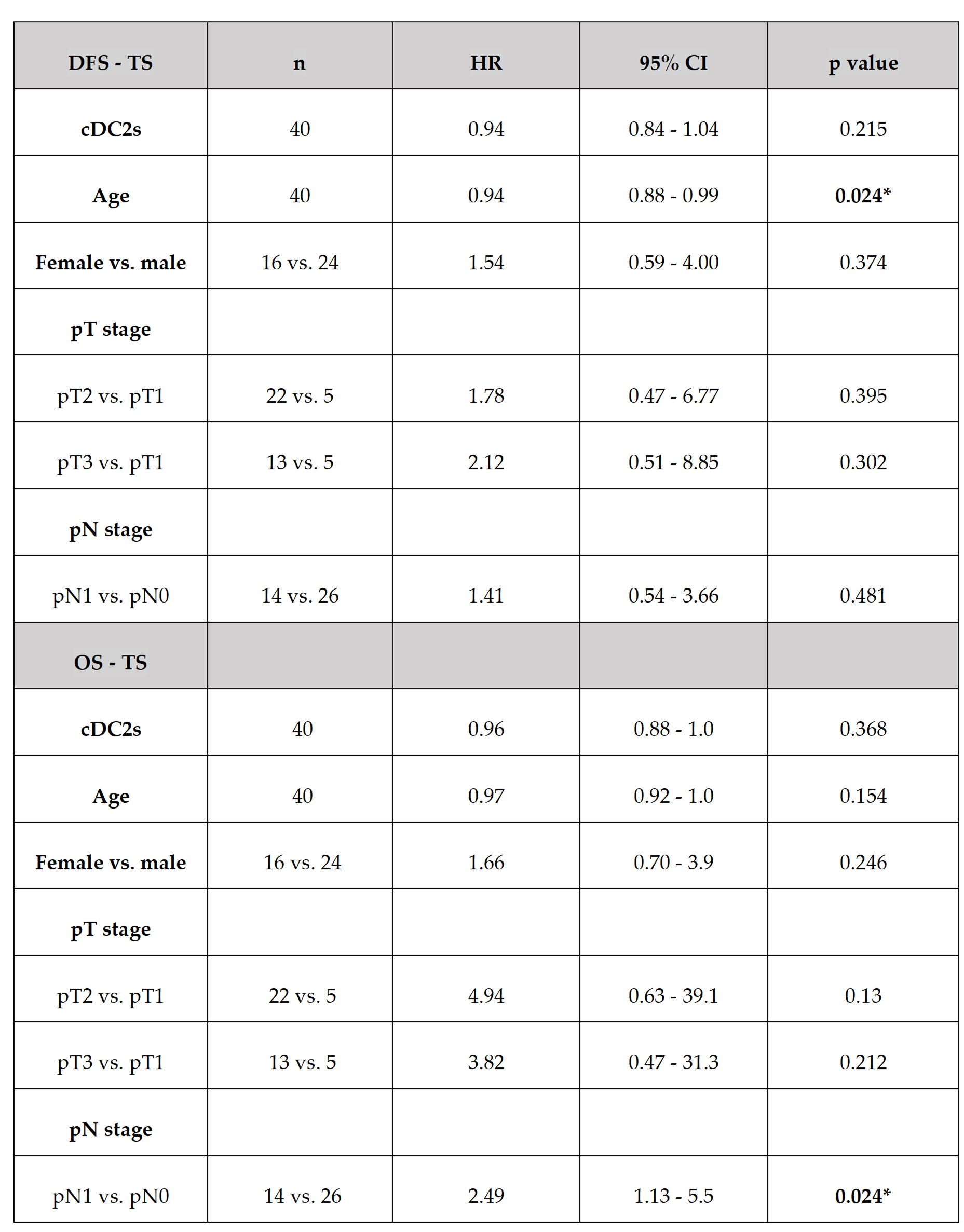

Supplement: Supplementary file 1 [file cancers-14-01216-s001.zip › Table S4.tif]

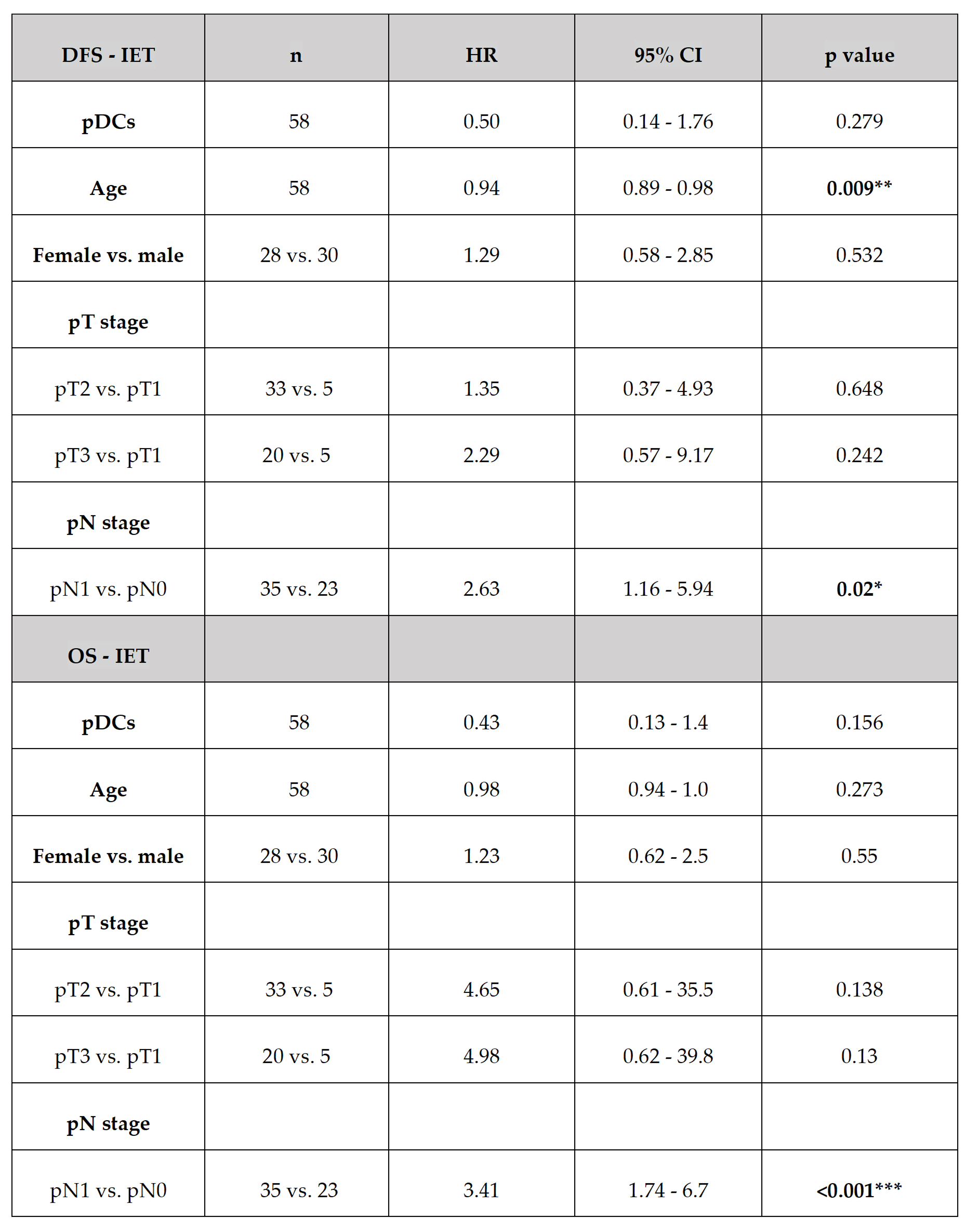

Supplement: Supplementary file 1 [file cancers-14-01216-s001.zip › Table S5.tif]
